# Microalbuminuria and left ventricular hypertrophy among newly diagnosed black African hypertensive patients: a cross sectional study from a tertiary hospital in Uganda

**DOI:** 10.1186/s13104-015-1156-2

**Published:** 2015-05-14

**Authors:** Juliet Nabbaale, Davis Kibirige, Emmanuel Ssekasanvu, Elias S Sebatta, James Kayima, Peter Lwabi, Robert Kalyesubula

**Affiliations:** Uganda Heart Institute, Mulago Hospital, P.O. Box 7051, Kampala, Uganda; Department of Medicine, Uganda Martyrs Hospital Lubaga, Kampala, Uganda; Department of Medicine, Makerere University College of Health Sciences, Kampala, Uganda; Department of Physiology, Makerere University College of Health Sciences, Kampala, Uganda

**Keywords:** Microalbuminuria, left ventricular hypertrophy, Left ventricular mass index, Newly diagnosed hypertensive patients, Cross sectional study, Africa, Uganda

## Abstract

**Background:**

Microalbuminuria is an early manifestation of kidney damage and independently predicts cardiovascular disease (CVD). Left ventricular hypertrophy (LVH) is also an early marker of cardiac manifestation of target organ damage among hypertensive patients. The prognostic significance of microalbuminuria and its correlation with left ventricular hypertrophy has not been extensively studied in African adult hypertensive populations. This study aimed at determining the prevalence of microalbuminuria, LVH in patients with microalbuminuria and the correlation between microalbuminuria and LVH among newly diagnosed black adult hypertensive patients attending a large outpatient hypertension clinic or admitted on the cardiology ward at Mulago national referral and teaching hospital and Uganda Heart Institute in Kampala, Uganda.

**Methods:**

In this cross-sectional study, 256 newly diagnosed eligible black adult hypertensive patients attending the outpatient hypertension clinic or admitted on the cardiology ward at Mulago national referral and teaching hospital and the Uganda Heart Institute, Kampala Uganda were consecutively recruited over a period of 5 months. Data on socio-demographics, clinical and laboratory findings of the study participants was collected using a pre tested questionnaire. Two spot urine samples were collected to assess for microalbuminuria. Echocardiography (ECHO) was done to assess for the left ventricular mass index using the formula of Teicholz as evidence for early hypertensive heart disease.

**Results:**

The mean age/standard deviation of the study participants was 54.3 ± 6.2 years with a female predominance (162, 63.3 %). The prevalence of microalbuminuria among newly diagnosed hypertensive patients was 39.5 %. The prevalence of LVH among patients with microalbuminuria was found to be 17 %. There was a positive correlation between microalbuminuria and left ventricular hypertrophy among the newly diagnosed adult hypertensive patients at Mulago Hospital (r = 0.185, p = 0.003).

**Conclusions:**

This study demonstrates that microalbuminuria is highly prevalent among newly diagnosed black hypertensive patients and in the presence of LVH. There is also a positive correlation between microalbuminuria and LVH among newly diagnosed hypertensive patients. Since it is a less costly and readily available test, it can be used to predict presence of LVH especially in resource limited settings where ECHO services are not readily available.

## Background

Recent evidence demonstrates that cardiovascular diseases (CVD) are the commonest cause of deaths globally. Low- and middle-income countries are disproportionally affected, with over 80 % of CVD deaths occurring in these countries [1, 2]. Hypertension (HT) is a growing public health problem and it is now being widely reported in many rural and urban parts of Sub Saharan Africa (SSA) as one of the commonest cause of morbidity and mortality [3].

The reasons for this growing burden are multiple, ranging from socio-economic changes and genetic influence. At a genetic level, there is growing evidence showing an association between elevated diastolic BP and CaMK4 affecting endothelial functions like controlling vascular resistance hence increasing the risk of HT [4].

Recently, a burgeoning burden of HT has been described both in rural and urban areas of Uganda. One earlier community based study conducted in the former Teso district in 1941 reported a very low frequency of HT of 2.9 % among adults aged 21-50 years [5]. Another similar population based study was conducted in the early 1960’s in Kasangati, a rural community outside of Kampala city revealed prevalence of 13.7 % [6]. Recent similar studies have documented age standardized HT prevalence to range between 14.6 % to 30.5 % [7–10]. In one rural study, a pre HT, an antecedent to clinically detected HT was reported to occur in 33.9 % of the study participants [9].

Microalbuminuria is an early indicator of renal damage and has been demonstrated as one of the principal predictive factors of cardiovascular (CV) complications, all cause and cardiovascular mortality independent of the traditional risk factors like dyslipidemia, hypertension [11, 12]. Left ventricular hypertrophy (LVH) determined either by standard 12-lead electrocardiography (ECG) or echocardiography is also a marker of subclinical organ damage related to hypertension and an independent predictor of cardiovascular morbidity/mortality [13].

Regression of microalbuminuria and LVH with standard anti hypertensive therapy (mainly with angiotensin converting enzyme inhibitors) is associated with a significant reduction in the associated CV morbidity and mortality [14]. Beta blockers especially the cardio selective ones also possess a role in LVH regression, albeit a minor role [15]*.*

There is paucity of studies on the burden of microalbuminuria and its correlation with LVH among black African newly diagnosed adult HT patients in Uganda. This study therefore aimed at examining the burden of microalbuminuria and its correlation with LVH in newly diagnosed adult HT patients attending the outpatient hypertension clinic or admitted on the cardiology ward at Mulago national referral and teaching hospital and the Uganda Heart Institute, Kampala Uganda.

## Methods

### Study design and setting

This cross sectional study was carried out in the outpatient hypertension clinic, the cardiology ward of Mulago national referral and teaching hospital, Kampala Uganda and the Uganda Heart Institute, Kampala Uganda. Mulago hospital serves as the national referral hospital and university teaching hospital for the Makerere University College of Health Sciences. It has a 1500 bed capacity.

### Study population and sampling technique

The study population comprised of 256 newly diagnosed eligible adult hypertensive patients attending the outpatient hypertension clinic or admitted on the cardiology ward at Mulago national referral and teaching hospital and the Uganda Heart Institute in Kampala, Uganda. The inclusion criteria into the study were: newly diagnosed adult hypertensive patients (aged ≥18 years of age) and those who gave written informed consent to participate in the study.

**Fig. 1:**
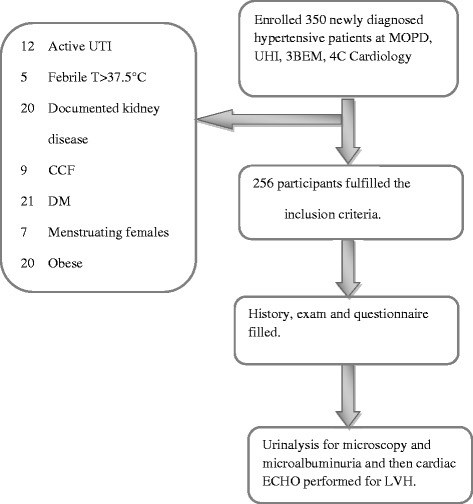
Patient flow chart on recruitment

Patients with documented chronic kidney disease, catheterization, fever (temperature above 37.5 °C), congestive cardiac failure, female patients who were menstruating, active urinary tract infection, patients with diabetes mellitus, those taking ACEIs, obese patients and those who had been under vigorous exercise in the past 24 h were excluded from the study (Fig. 1). Vigorous exercise was defined as that which could cause sweating, heavy breathing or fast heart beat like brisk walking in our African context.

They were consecutively recruited over a period of 5 months (June to October 2012) until the calculated sample size was attained.

#### Sample size calculation

Using prevalence of 21.3 % of microalbuminuria among newly diagnosed hypertensive patients reported by Rayner B et al in South Africa in 2006 [16], with an 80 % power and a two sided α < 0.05, a sample size of 256 was estimated using the Kish Leslie (1965) formula.

#### Data collection

A pre tested questionnaire was used to collect the socio-demographic and clinical data of the study participants. Blood pressure was measured using standard procedures using a standard Manual Dekamet Accoson MK3® mercury sphygmomanometer machine. Using standard methods, anthropometric measurements of weight in kilograms (kg) and the height in meters (m) were also performed and body mass index (BMI) calculated using the formula, BMI = weight in kg/height in m2.

### Assessment of microalbuminuria

The study participants were first instructed about the process of collecting a midstream urine sample. They were provided with two urine containers and requested to provide two samples of about 10 mls of mid-stream urine. One sample was collected in a sterile bottle for urinalysis including microscopy of the urine sediment. The second one was collected in a urine container with a preservative called 0.2 % sodium-azide for measuring of urinary albumin. The urine samples were sent to the Uganda Heart Institute laboratory for analysis which was done by the Immuno-turbidimmetric Method (RANDOX). Microalbuminuria was defined as a random urine albumin level between 30 and 299 mg/dl.

#### Echocardiography (ECHO) performance for assessing LVH

A trans thoracic ECHO was done by an experienced cardiologist with experience in echocardiography and clinical cardiology using a GE Vivid 7 echocardiography machine LV mass index was calculated using Teicholz formula where increased LV Mass index was values > 125 g/m^2^.

#### Data analysis

Data was coded and double entered in EpiData, cleaned and exported to STATA10 for analysis. Descriptive analysis of the study population was conducted. Pearson chi-square *X*^2^ and odds ratio analysis was applied to determine strength of association for categorical risk factors. A p-value of less than 0.05 was considered significant.

Association between microalbuminuria, LVH and the study variables were compared to test the strength of the association between them and the variables that included age, sex, education level, marital status and occupation. The prevalence of microalbuminuria was determined using proportions. The correlation between microalbuminuria and LVH was determined using Spearman’s rank correlation analysis technique.

#### Ethical consideration

This study was approved by the department of medicine, College of Health Sciences Makerere University and the research and ethics committee of the School of Medicine, Makerere University Kampala Uganda. All the study participants provided written informed consent to participate in this study.

## Results

### Socio demographic characteristics of study participants

The mean age/standard deviation of the study participants was 54.3 ± 6.2 years with a female predominance (162, 63.3 %). The age range of the study participants was from 17 to 94 years with majority aged between 35–54 years (43.0 %) and 55–74 years (33.2 %). Table 1 and Fig. 1 summarize the socio demographic characteristics and the flow chart of the study participants respectively.

**Table 1:** Socio-demographic characteristics of study participants

Characteristics	Frequency	Percentage (%)
Gender		
Female	162	63.3
Male	94	36.7
Age		
18 – 34	34	13.3
35 – 54	110	43
55 – 74	85	33.2
75 – 94	27	10.5
Level of education		
No Formal education	42	16.4
Primary education	88	34.4
Secondary education	66	25.8
Tertiary education	60	23.4
Marital Status		
Single	30	11.7
Married	165	64.5
Divorced	8	3.1
Cohabiting	1	0.4
Separated	13	5.1
Widowed	39	15.2

#### Prevalence of microalbuminuria and LVH

The overall prevalence of microalbuminuria and LVH among the study participants was 39.5 % and 14.1 % respectively. The age adjusted prevalence of microalbuminuria (31.7 %) was lower compared to the unadjusted prevalence as shown in Table 2.

**Table 2:** Age-adjusted prevalence of microalbuminuria in age groups

Age	Frequency (N)	Non–age adjusted (%)	Age-adjusted prevalence (%)
18–34	34	12	1.6
35–54	110	42	18
55–74	85	32	10.6
75–94	27	14	1.48
Total	256	100	31.7 %

According to the different categories of HT, 72 %, 8 % and 2 % of the study participants with controlled HT, stage 1 and stage 2 HT had microalbuminuria respectively. LVH was highly prevalent among the patients with stage 2 hypertension (85 %) and controlled HT (12 %). Only 12 % of the participants who had stage 1 hypertension had LVH.

#### Prevalence of LVH among study participants with microalbuminuria and according to age categories

The prevalence of LVH among the study participants with microalbuminuria was 17 %, higher than the overall study prevalence of LVH of 14.1 % (shown in Table 3). LVH was more prevalent in the older age groups of 55–74 (15, 41.7 %) and 75–94 years (6, 16.7 %) compared to the younger age groups (Table 4).

**Table 3:** Prevalence of LVH among participants with microalbuminuria

		LVH		Total
		Present	Absent	
Microalbuminuria	Present	17 (17.0 %)	83 (83.0 %)	100 (39.5 %)
	Absent	19 (12.2 %)	137 (87.8 %)	156 (60.5 %)
Total		36 (14.1 %)	220 (85.9 %)	256 (100 %)

**Table 4:** Prevalence of LVH among different age categories

LVH		15–34	35–54	55–74	75–94	Total
Present	Frequency	2 (5.6 %)	13 (36.1 %)	15 (41.7 %)	6 (16.7 %)	36 (14.1 %)
Absent	Frequency	32 (14.5 %	97 (44.1 %)	70 (31.8 %)	21 (9.6 %)	220 (85.9 %)
Total		34	110	85	27	256

#### Prevalence microalbuminuria and LVH according to BMI

Microalbuminuria was detected in 72.3 %, 18.2 % and 9.1 % of the study participants with a normal BMI, under weight and overweight respectively. LVH occurred in 66.7 %, 19.4 % and 13.9 % of study participants with normal BMI, underweight and overweight respectively.

#### Prevalence of microalbuminuria and LVH across stages of HT (Major revision 1)

Seventy two percent (72 %) of the study participants with controlled hypertension had microalbuminuria, while 8 % of those who had stage 1 hypertension had microalbuminuria. Only 2 % of tho study participants who had stage 2 hypertension had microalbuminuria. Sixty eight (68 %) of the study participants who had controlled hypertension had LVH while 12 % of the participants who had stage 1 hypertension had LVH and 85 % of the participants in stage 2 hypertension had LVH.

#### Prevalence of hypertensive heart failure clinically and by echocardiography

52.3 % of the study participants had diastolic heart failure and 18.7 % of the study participants had systolic heart failure on echocardiography. However, 46.7 % of the same study participants had Class 1 NYHA symptoms, 24.1 % were in class 11, 9.2 % were in class 111 and only 2 % of the study participants were in class 1 V.

#### Correlation between microalbuminuria and LVH among study participants

**Fig. 2:**
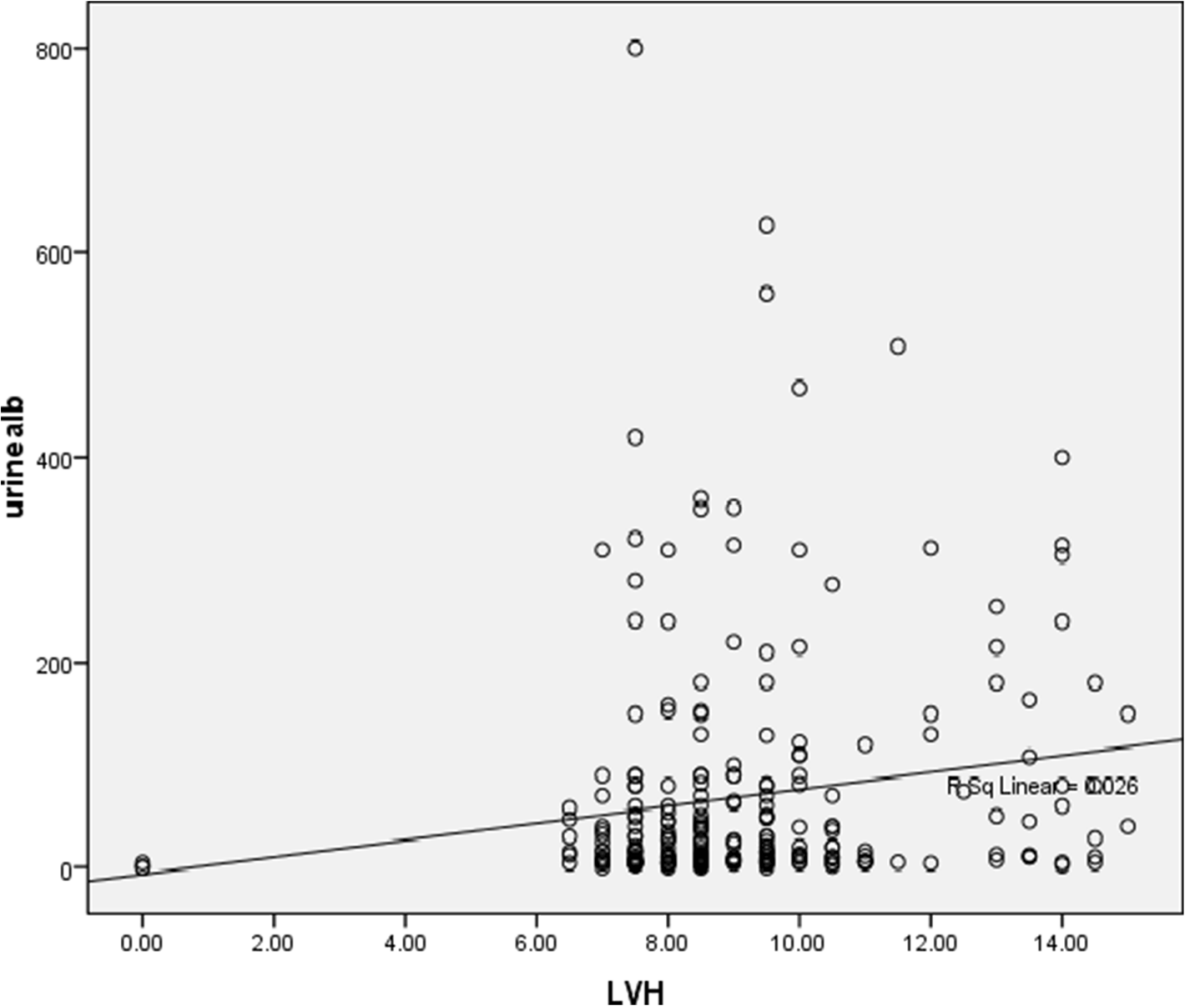
Scatter plot showing the correlation between microalbuminuria and LVH

As shown in Table 5, there was a positive correlation between microalbuminuria and LVH (r = 0.185, p = 0.003). This close association is further illustrated by the bivariate scatter plot (Fig. 2).

**Table 5:** Correlation between microalbuminuria and LVH

			Microalbuminuria	LVH
Spearman's rank correlation	Microalbuminuria	Correlation Coefficient	1.000	0.185
		p-value		0.003
		N	256	256
	LVH	Correlation Coefficient	0.185	1.000
		p-value	0.003	
		N	256	256

## Discussion

To our knowledge, this is the first study to assess the burden of microalbuminuria and its correlation with LVH among newly diagnosed adult HT patients in Uganda, a country with a growing burden of CVD. It unequivocally illustrates a high overall prevalence of microalbuminuria and LVH of 39.5 % and 14.1 % respectively, both features of target organ damage among the study participants in Uganda. These proportions represent a significant group of newly diagnosed adult hypertensive individuals who are at an increased risk of CV morbidity and mortality. The study also noted a positive correlation between LVH and microalbuminuria.

The reported prevalence of microalbuminuria in our study is comparable to what has been reported in one large international multi-center study [17] and in other African hypertensive populations [16, 18, 19]. In the international, observational, practice-based study i-SEARCH (Survey for Evaluating Microalbuminuria Routinely by Cardiologists in patients with Hypertension) that was designed to assess the frequency with which microalbuminuria occurred in a large outpatient population of 21,050 patients who were currently treated or newly diagnosed with hypertension and were under professional care in 26 countries, a high overall prevalence of microalbuminuria of 58.4 % noted [17].

Microalbuminuria was significantly related to the presence of specific predictors, including male gender, abnormally high waist circumference, increased blood pressure levels (systolic ≥120 mmHg, diastolic ≥100 mmHg), creatinine clearance ≥50 ml/min, or clinical conditions such as diabetes mellitus, congestive heart failure, history of cerebral pathology, and peripheral arterial disease.

Among African hypertensive populations, in a study performed in Morocco that was part of the global study described above (i-SEARCH study), the prevalence of microalbuminuria among the 476 patients recruited from 40 cardiology centers was 67.8 % [18]. This high prevalence in the Moroccan study compared to the one documented in the global study (58.4 %) could probably be explained by the high proportion of patients with suboptimal blood pressure control (81.4 %) and presence of multiple co-existing CV risk factors like diabetes mellitus, dyslipidemia and obesity which influence microalbuminuria (≥65 % of the patients had ≥3 CV risk factors).

In another similar study done in Nigeria, the prevalence of microalbuminuria in 96 newly diagnosed non diabetic hypertensive patients was 32.3 % compared to 6.3 % in age and sex matched controls [19]). In a multi racial South African study among 1901 ambulatory hypertensive patients, the overall prevalence of microalbuminuria was 21.3 % [16]). The independent predictors of microalbuminuria were severe hypertension (OR 2.9, p < 0.0001), type 2 diabetes (OR 2.5, p < 0.002), and Asian ethnic group (OR 2.0, p = 0.02).

LVH in our study was documented in 14.1 % of the study participants, a finding comparable to that in the South African study (18.9 %) [16]. In our study, LVH was also more frequent in patients with microalbuminuria compared to those without (17 % vs. 12.2 %). A higher frequency of LVH in patients with microalbuminuria has also been described in other similar studies [19–21].

The correlation between microalbuminuria and LVH in our study was found to be positive with a correlation coefficient of 0.185 and p value of 0.003. Similarly, microalbuminuria has been shown to positively correlate with LVH in other large studies [22, 23] and other cardiac abnormalities like left ventricular dysfunction and ischemic heart disease [24].

Microalbuminuria as a marker of early renal damage reflects widespread vascular damage (microangiopathy), a pro inflammatory state (with increased levels of IL-6, TNF-α, CRP and fibrinogen) and ensuing endothelial dysfunction. In addition to hypertension, microalbuminuria can also result from other unfavorable metabolic profile like hyperinsulinaemia and dyslipidemia [11, 12]. This hence augments development and progression of atherosclerotic disease.

With advancing stages of HT, it has been illustrated that both microalbuminuria and LV mass increase with worsening BP levels. LVH as a marker of target organ damage and a well known predictor of CV events has an independent association with a pro inflammatory state, and in particular elevated fibrinogen levels, which is associated with development of atherosclerosis [25].

Other than persistently increased volume or pressure overload on the left ventricle as occurs in HT, LVH can develop in aortic stenosis, hypertrophic cardiomyopathy and physiological LVH as seen in athletes.

## Conclusions

This study demonstrates a high prevalence of microalbuminuria and LVH, predictors of CV morbidity and mortality in this patient subgroup in Uganda and a corresponding positive correlation between both conditions.

Screening for urine albumin excretion should be extensively adopted in clinical care as a relatively simple, readily available and cheap process to facilitate early vascular disease detection as a strategy of averting HT related complications like LVH and optimal management in Uganda and SSA.

### Study limitations

Microalbuminuria was assessed on a single occasion using a spot urine sample although guidelines recommend triple testing (2 out of 3 tests need to be positive). However, it has been suggested that diagnosis of microalbuminuria can be more simply achieved by using an efficient and accurate method of taking a spot urine sample or an early morning urine specimen to minimize changes that occur during the day, this method does not require any special preparation prior to collecting the urine sample. However, 24-h and overnight urine collections are time consuming and often inaccurate particularly because incomplete collections are frequent. Vigorous exercise in 24 h preceding the albuminuria test could have increased the prevalence in our study patients however there was an attempt to assess this cause, albeit subjectively*.*

## Abbreviations

BMI: Body mass index (BMI) which is calculated using the formula BMI = weight in kg/height in m2.
CVD: Cardiovascular disease.
CV: Cardiovascular.
ECHO: Echocardiography.
ECG: Electrocardiography.
HT: Hypertension.
IVS: Inter ventricular septal thickness.
LVH: Left ventricular hypertrophy.
LVPW: Left ventricular posterior wall thickness.
SSA: Sub Saharan Africa.

## References

[1] Santulli G (2013). Epidemiology of Cardiovascular Disease in the 21st Century: updated numbers and updated fact. JCvD.

[2] WHO/Cardiovascular diseases. http://www.who.int/mediacentre/factsheets/fs317/en/ accessed 26 January 2015.

[3] Hendriks M, Wit F, Roos M, Brewster L, Akande T, de-Beer I (2012). Hypertension in Sub-Saharan Africa: Cross-Sectional Surveys in Four Rural and Urban Communities. PLoS One.

[4] Santulli G, Cipolletta E, Soriento D, Del Giudice C, Anastasio A, Monaco S (2012). CaMK4 Gene Deletion Induces Hypertension. J Am Heart Assoc.

[5] Williams A (1941). The Blood Pressure of Africans. East African Med J.

[6] Shaper AG (1969). Blood pressure and body build in a rural community in Uganda. East Afr Med J.

[7] Maher D, Waswa L, Baisley K, Karabarinde A, Unwin N, Grosskurth H (2011). Distribution of hyperglycaemia and related cardiovascular disease risk factors in low-income countries: a cross-sectional population-based survey in rural Uganda. Int J Epidemiol.

[8] Wamala J, Karyabakabo Z, Ndungutse D, Guwatudde D (2009). Prevalence factors associated with Hypertension in Rukungiri District, Uganda - A Community-Based Study. Afr Health Sci.

[9] Nuwaha F, Musinguzi G (2013). Pre-hypertension in Uganda: a cross-sectional study. BMC Cardiovasc Disord.

[10] Kotwani P, Kwarisiima D, Clark T, Kabami J, Geng E, Jain V (2013). Epidemiology and awareness of hypertension in a rural Ugandan community: a cross-sectional study. BMC Public Health.

[11] Abdelhafiz A, Ahmed S, El-Nahas M (2011). Microalbuminuria: Marker or Maker of Cardiovascular Disease. Nephron Exp Nephrol.

[12] Stehouwer C, Smulders Y (2006). Microalbuminuria and risk for cardiovascular disease: analysis of potential mechanisms. J Am Soc Nephrol.

[13] Bombelli M, Facchetti R, Carugo S, Madotto F, Arenare F, Quarti-Trevano F (2009). Left ventricular hypertrophy increases cardiovascular risk independently of in-office and out-of office blood pressure values. J Hypertens.

[14] Hsieh B (2005). Prognostic value of electrocardiographic criteria for left ventricular hypertrophy. Am Heart J.

[15] George T, Ajit M, Abraham G (2010). Beta blockers and left ventricular hypertrophy regression. Indian Heart J.

[16] Rayner B, Becker P (2006). The prevalence of MA and ECG LVH in hypertensive patients in private practices in South Africa. Cardiovasc J South Afr.

[17] Böhm M, Thoenes M, Danchin N, Reil J, Volpe M (2008). Overview of the i-SEARCH Global Study: cardiovascular risk factors and microalbuminuria in hypertensive individuals. High Blood Press Cardiovasc Prev.

[18] Habbal R, Sekhri A, Volpe M (2010). Prevalence of microalbuminuria in hypertensive patients and its associated cardiovascular risk in clinical cardiology: Moroccan results of the global i-SEAR CH survey - a sub-analysis of a survey with 21 050 patients in 26 countries worldwide. Cardiovasc J Afr.

[19] Busari O, Opadijo G, Olarewaju T, Omotoso A, Jimoh A (2010). Electrocardiographic correlates of microalbuminuria in adult Nigerians with essential hypertension. Cardiol J.

[20] Forlemu A, Menanga A, Ashuntantang G, Kingue S (2013). Urinary Protein Excretion Is Associated with Left Ventricular Hypertrophy in Treatment- Naïve Hypertensive Patients in an African Hospital Setting. Cardiorenal Med.

[21] Hitha B, Pappachan J, Pillai H, Sujathan P, Ramakrishna C, Jayaprakash K (2008). Microalbuminuria in Patients with Essential Hypertension and its Relationship to Target Organ Damage: An Indian Experience. Saudi J Kidney Dis Transpl.

[22] Pontremoli R, Ravera M, Bezante G, Viazzi F, Nicolella C, Berruti V (1999). Left ventricular geometry and function in patients with essential hypertension and microalbuminuria. J Hypertens.

[23] Wachtell K, Palmieri V, Olsen M, Bella JN, Aalto T, Dahlöf B (2002). Urine albumin/creatinine ratio and echocardiographic left ventricular structure and function in hypertensive patients with electrocardiographic left ventricular hypertrophy: The LIFE study. Losartan Intervention for Endpoint Reduction. Am Heart J.

[24] Liu J, Robbins D, Palmieri V (2003). Association of albuminuria with systolic and diastolic left ventricular dysfunction in type 2 diabetes: The Strong Heart Study. J Am Coll Cardiol.

[25] Palmieri V, Celentano A, Roman J, de-Simone G, Lewis M, Best L (2001). Fibrinogen and preclinical echocardiographic target organ damage: the Strong Heart Study. Hypertension.

